# The 2^nd^ edition of the Romanian National Neurology Forum: from idea to implementation in the health system – here, now, together!

**DOI:** 10.25122/jml-2024-1003

**Published:** 2024-02

**Authors:** Dafin Fior Mureşanu, Diana Chira, Ştefana-Andrada Dobran, Alexandra Gherman

**Affiliations:** 1 RoNeuro Institute for Neurological Research and Diagnostic, Cluj-Napoca, Romania; 2 Department of Neuroscience, Iuliu Hatieganu University of Medicine and Pharmacy, Cluj-Napoca, Romania

## THE 2^ND^ EDITION OF THE ROMANIAN NATIONAL NEUROLOGY FORUM: IMPROVING THE HEALTHCARE SYSTEM

**Figure 1:**
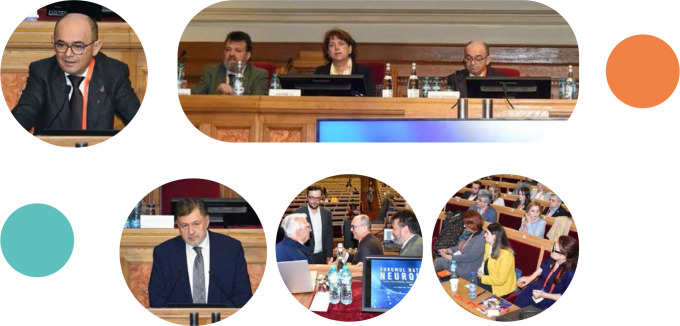
The Second National Neurology Forum 2024: First row (from left to right) - Prof. Dr. Dafin F. Muresanu (president of the Neurology Commission for the Ministry of Health), Prof. Dr. Bogdan O. Popescu (vice-rector of Carol Davila University of Medicine and Pharmacy in Bucharest), Prof. Dr. Cristina Tiu (president of the Romanian Society of Neurology); second row - Prof. Dr. Alexandru Rafila (Minister of Health); Prof. Dr. Victor Voicu (President of Medical Science Section of Romanian Academy), CSII Stefan Strilciuc (Director of the Research Center for Functional Genomics, Biomedicine and Translational Medicine at the Iuliu Hatieganu University of Medicine and Pharmacy Cluj-Napoca), Prof. Dr. Dafin Muresanu, and Prof. Dr. Bogdan O. Popescu; photos of the participants.

The 2024 Romanian National Neurology Forum (NNF), a hybrid event held in **Bucharest**, between **18-19 April** (the first edition of the NNF took place between 21-22 April 2023, at the Palace of Parliament, in Bucharest), at the **Palace of the Patriarchate**, was set to complement the Annual Congress of the Romanian Society of Neurology, bringing together strategic insights and perspectives on health policies for implementing systemic reforms in the field. Themes regarding patient perspectives, medical practice, and public policy converged and provided the event with a platform designed to outline necessary steps for enhancing the Romanian healthcare system. The dynamics of the NNF characterized by highly interactive discussions, networking sessions, and specific workshops allowed the Forum to bring together over 300 professionals (Figure 1) and bridge strategic visions with public policy perspectives as a next step towards building stronger and more resilient health systems adapted to the needs of the population.

This year’s edition of the Forum reunited experts in neurology, cardiology, neurosurgery, diabetes and nutritional disorders, physical and rehabilitation medicine, and many more. The event was organized by Prof. Dr. Dafin Muresanu, president of the Neurology Commission for the Ministry of Health, Prof. Dr. Cristina Tiu, president of the Romanian Society of Neurology, and Prof. Dr. Bogdan O. Popescu, vice-rector of Carol Davila University of Medicine and Pharmacy, with the support of the Foundation of the Society for the Study of Neuroprotection and Neuroplasticity, Ministry of Health, Romanian Society of Neurology, Carol Davila University of Medicine and Pharmacy, Iuliu Hatieganu University of Medicine and Pharmacy, and George Emil Palade University of Medicine, Pharmacy, Science and Technology of Targu Mures.

Well-known and appreciated plenary debates, intensive thematic workshops, and a course for clinicians offered stakeholders the requisites to brainstorm tangible solutions for the Romanian healthcare system. The main drive of the event, the National Strategy for Cardiovascular and Cerebrovascular Diseases (SNBCC), aligns with the 2023–2030 National Health Strategy and European initiatives to provide a comprehensive reform plan to improve the health status of the Romanian population. The strategy addresses key areas such as prevention, infrastructure, access to neurorehabilitation, medical staff training, and improving the national network for acute stroke care to ultimately enhance patient outcomes, quality of life, and community reintegration. SNBCC targets equitable services centered upon the needs of the individual, the family and caregivers, and the community through concrete action supported by multidisciplinary evidence-based approaches that offer particular emphasis to vulnerable groups.

## NEUROLOGICAL HEALTHCARE IN ROMANIA

The Romanian healthcare system is challenged by an increasing prevalence of neurological diseases, such as stroke, epilepsy, multiple sclerosis, Parkinson’s disease, migraine, and rare neurological diseases. These conditions can profoundly impact the patients’ and caregivers’ lives and significantly burden the healthcare system, leading to increased mortality and morbidity, higher direct and indirect costs, and decreased quality of life.

The rising prevalence of neurological conditions reflects the global trend linked to factors such as population ageing, sedentary lifestyles, unhealthy diets, and shortcomings in the healthcare system.

It should be highlighted that in Romania, the significant challenges related to access to treatment and rehabilitation, resource distribution, infrastructure, prevention, awareness, and education in the general population call for comprehensive systematic approaches.

## OPTIMIZING NATIONAL CARE THROUGH TARGETED WORKSHOPS

**Figure 2:**
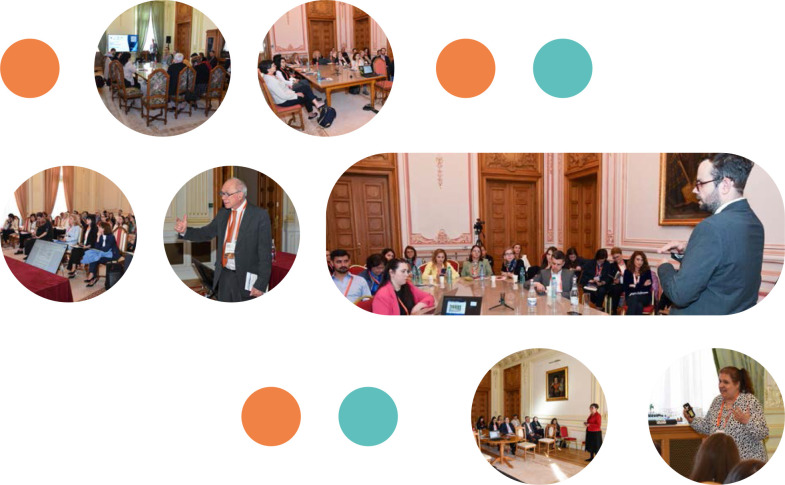
Photographs from the workshops (from left to right): first row - Rare neurological diseases workshop and Epilepsy workshop; second row - Parkinson’s disease workshop, Prof. Volker Hömberg (WFNR President) at the EFNR–WFNR Joint Teaching Course on Neurorehabilitation, Implementation of the National Cardiovascular and Cerebrovascular Disease Strategy workshop; third row - Multiple sclerosis workshop and Headache and migraine workshop.

A core trait of the Forum, the multidisciplinary workshops (Figure 2) offered the participants and coordinators the adequate platform to evaluate the present national landscape, identify needs, propose interventions, and underscore areas for improvement for the various fields of interest covered by the event. Key themes emerged from the discussions, including the need to enhance awareness and education, increase access to treatments, support the nationwide screening programs and implementation of patient registries. Also, significant and detailed discussions focused on the improvement of diagnosis and treatment guidelines, reducing the financial burden of neurological diseases, and advancing healthcare digitalization as a tool to educate and offer support to a larger population.

## IMPLEMENTATION OF THE NATIONAL CARDIOVASCULAR AND CEREBROVASCULAR DISEASE STRATEGY WORKSHOP

The workshop offered an integrative perspective on the comprehensive management of cardiovascular and cerebrovascular diseases (CCD).

The participants and coordinators underscored the necessity of a solid theoretical framework for prevention and highlighted the need for efficient multisectoral interventions. The integration of the RES-Q stroke registry in the national healthcare system and expanding data collection represent important steps in the management of acute stroke, while a national health program for interventions for cerebrovascular disorders can aid in improving the national delivery of care.

Neurorehabilitation helps patients in the process of recovering their physical and psychological health and ensures community reintegration. In this subfield, the participants and coordinators identified the needs for improving neurorehabilitation infrastructure, developing language therapy, advancing specialized education for doctors and nurses, as well as implementing monitoring tools to facilitate patient transition from acute care towards neurorehabilitation, steps that must be supported by major investments in the sector.

In conclusion, the workshop laid the groundwork for enhanced strategies in prevention, treatment, and rehabilitation - crucial for optimizing patient care.

## PARKINSON'S DISEASE WORKSHOP

The themes approached during the workshop were the diagnosis, treatment, and care of patients affected by Parkinson’s Disease (PD).

A focal point was the presentation of the new guidelines from the Romanian Society of Neurology for diagnosis and treatment. The necessity for standardized imaging interventions was highlighted, and the issue of outpatient neurologists’ distribution in Romania underscored the need for detailed mapping to improve access to healthcare. Further on, a simplified scheme was proposed to aid in the identification of patients with advanced PD, aimed at improving the patient pathway and reducing the financial burden for the healthcare system.

At the same time, the workshop focused on developing the current PD registry, the need to expand awareness campaigns, and the establishment of a unified patient community to empower individuals to share their voices and contribute to advocacy regarding PD.

## MULTIPLE SCLEROSIS WORKSHOP

The workshop emphasized the role of open dialogue and collaboration among all actors at central, regional, and local levels, to address the needs of patients with multiple sclerosis (MS). Resource distribution, treatment access, and patient support are all crucial factors when discussing MS.

A main concern was to reconfigure the current financing procedures to better sustain ongoing access to treatment for all patients. In the case of pediatric patients, improving legislation for off-label medicine and the use of interferon is a key factor in supporting their needs. To support the care of patients with MS, enhanced access to high-quality diagnostic tools, ensuring better national surveillance of patients, and supporting the use of cost-effective treatments are essential.

At the national level, there is a dire need to secure appropriate human resources, digitalize services, and expand the network of diagnosis centers, as well as update treatment protocols and focus on personalized care. The development of a national registry and strengthening the network of specialists through unofficial sub-specialization were also recommended as measures that can aid in better understanding and treating this affection.

This chronic neurological disease calls for an extensive approach based on both improved collaboration with authorities and resource management, as well as enhanced access to treatment for patients by simplifying bureaucracy.

## EPILEPSY WORKSHOP

This workshop focused on developing specialized services for patients with epilepsy and emphasized the need for equitable access to care. The session highlighted the importance of improving education and managing risk factors and brought forward tangible solutions to facilitate the early identification of high-risk patients and promote preventive measures.

The discussions stressed the need for access to essential investigations like electroencephalography (EEG), prolonged VideoEEG with sleep recording, MRI, and neurocognitive evaluations. Participants identified the challenge of suboptimal technical standards and staff education at medical centers performing these investigations, many of which are underfunded. The accreditation of these centers and the enhancement of outpatient services were also advocated for. Additionally, the development of Regional Epilepsy and EEG Centers was proposed to bring diagnosis, treatment, and monitoring up to international standards.

The workshop also emphasized the need for a national epilepsy registry to support research and rapid identification of patients eligible for certain therapies, through continuous education for neurologists and other health professionals, as well as accreditation of EEG technicians.

## HEADACHE AND MIGRAINE WORKSHOP

This workshop tackled the often underdiagnosed and undertreated issues of headaches and migraines and focused on enhancing awareness and improving management through education and collaboration with international patient associations. Discussions highlighted the essential role of nurses in patient education and therapy management and emphasized the importance of comprehensive training to efficiently assist patients in their lifestyle adjustments.

The development of a patient registry for those with frequent chronic migraines to collect detailed data and improve monitoring and interventions was proposed. The discussions also outlined the need for expanded educational programs and the development of mobile apps to support diagnosis and track disease progression.

The session concluded by underscoring the significance of specialized networks and centers for headache treatment to ensure ongoing medical education to enhance care for patients suffering from headaches and migraines.

## RARE NEUROLOGICAL DISEASES IN ROMANIA

This workshop addressed the challenges of rare neurological diseases at national level, both for adults and for pediatric patients. Discussions emphasized the need for screening programs to enable access to personalized treatments and highlighted the transformation of compassionate use programs into stable, funded national initiatives. The activity identified critical steps like reducing diagnosis times through EU-approved treatments and simplifying legislative processes for faster implementation.

Key issues included the expansion of neonatal screening to cover rare neurological diseases and ensuring the reimbursement of essential diagnostic tests. The importance of updating national registries and guidelines in line with European standards was discussed, with a call for expertise centers to join the European Reference Network for validation.

The session concluded with the value of enhancing access to new therapies and continuous medical education for healthcare professionals, patients and caregivers, underlining the need for multidisciplinary perspectives to improve care for patients with rare neurological diseases.

## EFNR–WFNR JOINT TEACHING COURSE ON NEUROREHABILITATION

This joint teaching course facilitated by experts from the European Federation of NeuroRehabilitation Societies (EFNR) and the World Federation for NeuroRehabilitation (WFNR) offered the platform to distinguished speakers from Germany, South Korea, Israel and Romania to share practical solutions for enhancing neurorehabilitation capacities at a national level.

A key focus was on the role of virtual reality in the rehabilitation process of stroke patients, by simulating complex scenarios and engaging patients in therapeutic activities within a controlled environment. The session also tackled recent advances in neuromodulation technology, as having significant potential in treating chronic neurological conditions and alleviating symptoms in complex cases of MS, PD,, and other neurodegenerative disorders. Topics like neurotrophic factors, the role of motivation, and innovative treatments with botulinum toxin as part of the neurorehabilitation process completed the session.

This joint training course was a valuable opportunity for neurorehabilitation professionals to explore technologies, share best practices, and discuss strategies to meet patient needs in a multidisciplinary approach.

## THE NATIONAL NEUROLOGY FORUM: NEW HORIZONS

As mentioned in the beginning, the second edition of the National Neurology Forum highlighted the role and impact of multidisciplinary collaboration in addressing medical, organizational, and public health issues in Romania. Bringing together doctors, researchers, industry leaders, national authorities and patient representatives, alongside international specialists, the scientific event reunited stakeholders and paved the way to identifying solutions to problems related to organizational efficiency, access to care, prevention, diagnosis, treatment, rehabilitation, cost-effectiveness and the overall quality of life for Romanian patients affected by neurological disorders.

The 2025 edition of the National Neurology Forum will take place in April and will continue its pursuit to bridge future improvements and developments related to patient care and health policies.

*Join us and be part of the journey towards improving the health of the Romanian population and building a future where accessible, high-quality neurological healthcare is available to everyone!*

